# PARP3 controls TGFβ and ROS driven epithelial-to-mesenchymal transition and stemness by stimulating a TG2-Snail-E-cadherin axis

**DOI:** 10.18632/oncotarget.11627

**Published:** 2016-08-26

**Authors:** Olga Karicheva, José Manuel Rodriguez-Vargas, Nadège Wadier, Kathline Martin-Hernandez, Romain Vauchelles, Najat Magroun, Agnès Tissier, Valérie Schreiber, Françoise Dantzer

**Affiliations:** ^1^ Poly(ADP-ribosyl)ation and Genome Integrity, Laboratoire d'Excellence Medalis, UMR7242, Centre National de la Recherche Scientifique/Université de Strasbourg, Institut de Recherche de l'Ecole de Biotechnologie de Strasbourg, 67412 Illkirch, France; ^2^ Laboratoire de Biophotonique et Pharmacologie, UMR7213, Centre National de la Recherche Scientifique/Université de Strasbourg, Faculté de Pharmacie, 67401 Illkirch, France; ^3^ EMT and Cancer Cell Plasticity, Laboratoire d'Excellence DevWeCan, Equipe labellisée Ligue Nationale Contre Le Cancer, Centre de Recherche en Cancérologie, UMR INSERM 1052 CNRS 5286, Centre Léon Bérard, F-69008 Lyon, France

**Keywords:** Poly(ADP-ribose) polymerase 3 (PARP3), EMT, TGFβ, ROS, stem cells

## Abstract

Several members of the Poly(ADP-ribose) polymerase (PARP) family are essential regulators of genome integrity, actively prospected as drug targets for cancer therapy. Among them, PARP3 is well characterized for its functions in double-strand break repair and mitotis. Here we report that PARP3 also plays an integral role in TGFβ and reactive oxygen species (ROS) dependent epithelial-to-mesenchymal transition (EMT) and stem-like cell properties in human mammary epithelial and breast cancer cells. PARP3 expression is higher in breast cancer cells of the mesenchymal phenotype and correlates with the expression of the mesenchymal marker Vimentin while being in inverse correlation with the epithelial marker E-cadherin. Furthermore, PARP3 expression is significantly upregulated during TGFβ-induced EMT in various human epithelial cells. In line with this observation, PARP3 depletion alters TGFβ-dependent EMT of mammary epithelial cells by preventing the induction of the Snail-E-cadherin axis, the dissolution of cell junctions, the acquisition of cell motility and chemoresistance. PARP3 responds to TGFβ-induced ROS to promote a TG2-Snail-E-cadherin axis during EMT. Considering the link between EMT and cancer stem cells, we show that PARP3 promotes stem-like cell properties in mammary epithelial and breast cancer cells by inducing the expression of the stem cell markers SOX2 and OCT4, by increasing the proportion of tumor initiating CD44^high^/CD24^low^ population and the formation of tumor spheroid bodies, and by promoting stem cell self-renewal. These findings point to a novel role of PARP3 in the control of TGFβ-induced EMT and acquisition of stem-like cell features and further motivate efforts to identify PARP3 specific inhibitors.

## INTRODUCTION

Epithelial-to-mesenchymal transition (EMT) is a transdifferentiation programme that is important for organogenesis in the developing embryo, tissue injury repair and cancer progression [1]. EMT is characterized by the reversible loss of epithelial characteristics coupled with the gain of mesenchymal properties, increased motility and the acquisition of chemoresistance in tumor cells [2]. There is also clear evidence that EMT confers cancer stem-like cell properties [3–5]. At the molecular level, EMT is mediated by the activation of EMT transcription factors such as Snail (encoded by *SNAI1* gene), the loss of cell junctions components such as E-cadherin (encoded by *CDH1*) and zona occludens protein 1 (encoded by *TJP1*) and the accumulation of mesenchymal markers such as Vimentin (encoded by *VIM*). Activation of EMT has been particularly studied in breast tumorigenicity. Genome-wide transcriptional profiling of a large set of human breast cancer cell lines used as experimental models, defined the existence of the basal B subgroup with enhanced invasive properties and a mesenchymal gene expression signature, distinct from the less aggressive luminal or mixed basal/luminal (basal A) subgroups with a predominantly epithelial gene expression signature [6]. At the clinical level, patients who develop breast cancer with a basal-like molecular profile and upregulation of EMT markers are known to have a poor clinical outcome [7]. Consequently, an important goal in the field is identifying factors or extracellular signals that promote EMT.

Among them, the multitasking cytokine TGFβ frequently elevated in the tumor microenvironment emerged as a driving force [8, 9]. Growing evidence reveal the importance of TGFβ and oxidative stress/reactive oxygen species (ROS) interplay in tumorigenesis and malignancy [10]. TGFβ is able to stimulate ROS production directly or by downregulating antioxidative systems [11]. Till, ROS have been found to increase TGFβ signaling and to activate latent TGFβ [10]. It is also increasingly apparent that TGFβ signaling mediates cell response to DNA damage and genome stability as a way to confer resistance to cancer therapeutics [12–14].

Several recent studies also implicate the type II tissue transglutaminase TG2 (*TGM2*) as a regulator of EMT. TG2 is enriched in multiple tumors and cancer stem cells, it was shown to stimulate EMT in various cell models, confer stemness traits and was associated with drug resistance [15–18]. Subsequent studies revealed that TG2 links TGFβ signaling, EMT and stem cell properties [19, 20].

Other reports have shown that Poly(ADP-ribose) polymerase 1 (PARP1) also serves in EMT although with distinct conclusions depending on the cellular context [21–23]. PARP1, the founding member of the PARP family (17 members), synthesizes polymers of ADP-ribose onto target proteins using NAD^+^ as a substrate [24, 25]. Due to the predominant function of PARP1 in DNA repair, its inhibition appeared as the strategy of choice in various forms of cancer to potentialize the cytotoxic action of chemotherapy and radiotherapy or to selectively kill tumors with dysfunctional repair [26–28]. However, most clinically used PARP1 inhibitors bind to the PARP catalytic domain well conserved among the PARP family members thereby revealing a lack of specificity and raising question about their adverse effects. A high priority is to elucidate the biological activities of the more recently identified PARP members. Among them, the DNA-dependent PARP3 was specified to have key functions in the repair of double-strand breaks through NHEJ, in class switch recombination, in telomere segregation and microtubule spindle formation during mitosis and in transcriptional regulation during neuronal development in the zebrafish [29–34].

What is not yet clearly defined is the biological interest of targeting PARP3 in cancer therapy. In this study, we examined the transcriptional and protein expression level of PARP3 in human breast cancer cell lines of distinct subtypes expressing various levels of EMT traits [6]. The general elevated expression of PARP3 associated with the mesenchymal phenotype inspired the idea to investigate its function in the EMT program and to delineate the molecular pathway it regulates.

## RESULTS

### PARP3 expression positively correlates with the mesenchymal phenotype in human breast cancer cell lines

**Figure 1:**
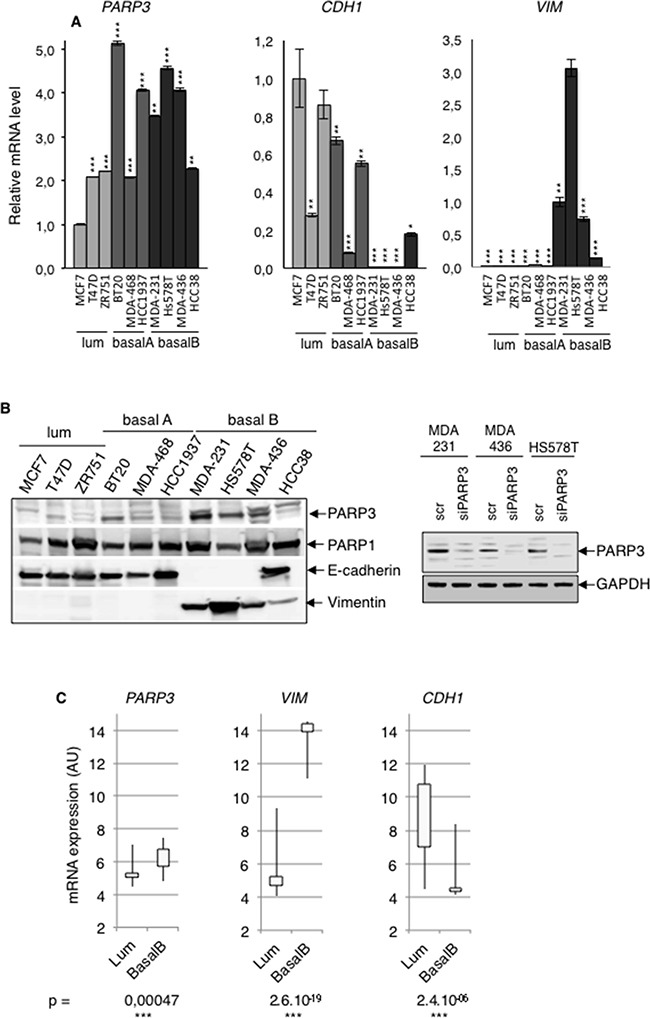
PARP3 expression is positively correlated with the mesenchymal phenotype in human breast cancer cells **A.** The mRNA expression levels of PARP3 (*PARP3)*, E-cadherin *(CDH1)* and Vimentin *(VIM)* were determined by RT-qPCR in various breast cancer cell lines of the luminal (MCF7, T47D, ZR751), basal A (BT20, MDA-MB468, HCC1937) or basal B (MDA-MB231, Hs578T, MDA-MB436, HCC38) subtypes. *TBP* mRNA was used for normalization. Error bars represent the mean (+/− s.d) of triplicates. *P<0,05, **P<0,01, ***P<0,001. Statistics were calculated on the differential expression in the different cell lines relative to MCF7 (*PARP3* and *CDH1*) and Hs578T (*VIM*). **B.** (Left) The protein expression levels of PARP3, PARP1, E-cadherin and Vimentin were examined on RIPA-like total protein extracts prepared as described in Material and methods and western blotting using the appropriate antibodies. Equivalent loading of protein extracts was verified by Coomassie staining (Supplementary Figure S1A). The PARP3 migrating band (nuclear PARP3) is indicated by the arrow and was confirmed by its absence upon siPARP3 in MDA-MB231, MDA-MB436 and Hs578T extracts (Right). The rabbit anti-PARP3 antibody (4698) detects nuclear PARP3 [29]. **C.** Relative mRNA transcript abundance of *PARP3*, *VIM* and *CDH1* in luminal and basal B human breast cancer cell lines according to the gene expression data set from the Cancer Cell line Encyclopedia (CCLE). Statistical values of the Pearson's correlation were determined according to Neve and collaborators [6].

In the course of our studies aimed to decipher the biological properties of PARP3, we compared the expression of PARP3 in a series of human breast cancer cell lines of distinct subgroups with predominantly luminal (luminal), mixed luminal/basal (basal A) and basal (basal B) features [6]. We found that PARP3 expression both at mRNA (Figure 1A) and protein levels (Figure 1B) was predominant in the mesenchymal-like basal B subtype while less or poorly detectable in the epithelial-like luminal subtypes. High levels of PARP3 mRNA and protein were often correlated with high expression of the mesenchymal marker Vimentin and loss of the epithelial marker E-cadherin. At the protein level, this correlation was specific for PARP3 and not observed for PARP1. In support of this initial observation, an extended analysis of the published *PARP3* gene expression profile in a larger panel of breast cancer cells from the Cancer Cell line Encyclopedia (CCLE) confirmed a significantly higher expression of *PARP3* in the basal B subtype displaying a *VIM*-high and *CDH1*-low gene expression signature compared to the luminal subtype (Figure 1C). Moreover, *PARP3* expression in these cell lines positively correlated with their EMT score (Supplementary Figure S1B). Collectively, these data suggested that *PARP3* is upregulated in breast cancer cell lines displaying a mesenchymal-like gene expression profile and raised the question of whether PARP3 might regulate the switch between the epithelial and mesenchymal phenotype. However, the stable ectopic expression of PARP3 in MCF10A or MCF7 cells was insufficient to spontaneously induce EMT associated alterations (Supplementary Figure S2).

### PARP3 expression is increased in the course of TGFβ-induced EMT

**Figure 2:**
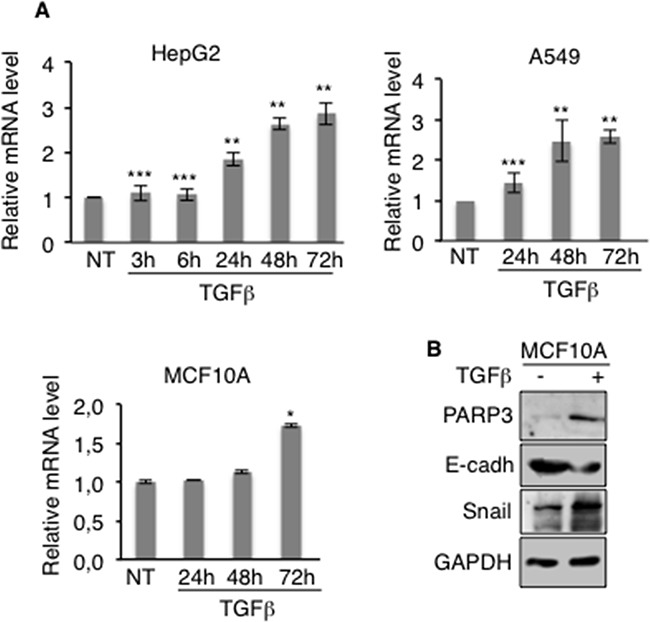
PARP3 expression is induced in the course of TGFβ-mediated EMT **A.** HepG2, A549 and MCF10A cells were mock-treated or incubated with TGFβ (2 ng/mL) for the indicated time points. The relative *PARP3* mRNA level was determined by RT-qPCR. *UBC* mRNA was used to normalise variability in template loading. Error bars represent the mean (+/− s.d) of triplicates. *P<0,05, **P<0,01, ***P<0,001 **B.** MCF10A were mock-treated or treated with TGFβ (2 ng/mL) for 72h. The protein expression levels of PARP3 and the EMT markers were determined by western blotting using the appropriate antibodies. GAPDH was used as a loading control.

EMT can be triggered by various growth and differentiation factors. Among them, TGFβ has emerged as a key regulator of EMT in late-stage carcinomas where it promotes invasion and metastasis [8, 9]. We therefore examined the effects of TGFβ on *PARP3* expression in different cell lines frequently used as models of inducible TGFβ-mediated EMT (Figure 2A). *PARP3* mRNA levels were increased in a time-dependent manner in the lung cancer cell line A549, the hepatocellular carcinoma cell line HepG2 and the mammary epithelial cell line MCF10A after TGFβ stimulation. MCF10A cells are routinely used to investigate TGFβ-induced EMT. We therefore analysed PARP3 protein levels in this model upon TGFβ treatment. We confirmed that PARP3 protein level was also increased in response to TGFβ in this model. Its upregulation correlates with the induction of the EMT master regulator Snail and the concomittant repression of the epithelial marker E-cadherin in response to TGFβ (Figure 2B). Based on these findings, we suggested that PARP3 might assist the EMT commitment of TGFβ-induced EMT.

### PARP3 promotes TGFβ-induced EMT, cell motility and chemoresistance in mammary epithelial cells

**Figure 3:**
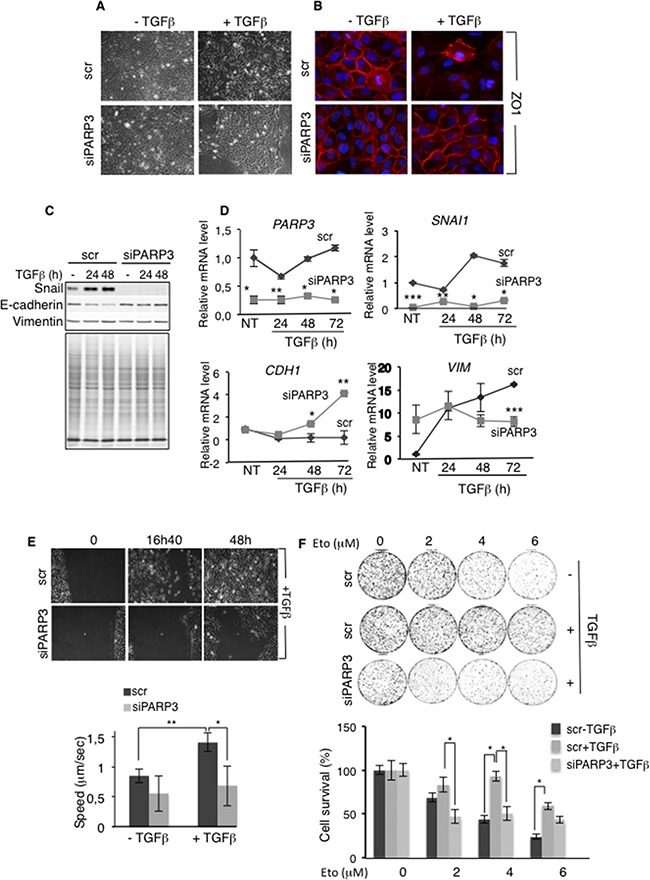
PARP3 silencing impairs TGFβ-induced EMT, migration and chemoresistance **A-D.** PARP3 silencing impairs TGFβ-mediated EMT. (A,B) Control (scr) and PARP3 depleted (siPARP3) MCF10A cells were either mock-treated or incubated with TGFβ (2 ng/mL) for 48h and analysed for EMT progression. (A) Morphological changes were monitored using phase contrast microscopy. (B) Immunofluorescence analysis of the presence and localization of the junction protein ZO1 (red). Nuclei were counterstained with DAPI. (C,D) Control (scr) and PARP3 depleted (siPARP3) MCF10A cells were either mock-treated or incubated with TGFβ (2 ng/mL) for the indicated time points. EMT markers were analysed (C) by western blotting using the appropriate antibodies. Coomassie staining of the protein extracts was used as loading control (D) or by RT-qPCR. *UBC* mRNA was used for normalization. Experiments were performed >3 times giving similar results. Error bars represent the mean (+/− s.d) of triplicates. Statistics were calculated on the differential expression between siPARP3 relative to scr. *P<0,05, **P<0,01, ***P<0,001. **E.** PARP3 silencing impairs TGFβ driven cell motility (scratch wound closure) (top) Light microscopy of representative areas of scratch assays on cultures of control (scr) or PARP3 silenced (siPARP3) MCF10A cells treated with TGFβ (2 ng/mL) throughout the experiment. (bottom) Analysis of video recording of the *in vitro* scratch-wounds. Error bars represent the mean (+/− s.d) of triplicate experiments. *P<0,05, **P<0,01 **F.** TGFβ confers enhanced resistance to etoposide that is reduced upon PARP3 silencing. Dose response clonogenic survival curves of control (scr) or PARP3 depleted (siPARP3) MCF10A cells mock-treated or treated with TGFβ (2 ng/mL) for 24h and exposed to increasing concentrations of etoposide. Experiments were performed 3 times. Mean values of triplicates (+/− s.d) are shown. *P<0,05.

To investigate this hypothesis, we silenced PARP3 in MCF10A cells using siRNA approach and analysed the impact on EMT characteristics promoted by TGFβ (Figure 3A–3D). TGFβ treatment of MCF10A cells resulted in EMT with transformation from a cobblestone-like epithelial morphology to an elongated fibroblast-like morphology (Figure 3A), dissolution of the ZO1-stained tight junctions (Figure 3B), upregulation of Snail and the concomitant repression of E-cadherin at both the mRNA and protein levels (Figure 3C–3D). In contrast, the mesenchymal marker Vimentin was only upregulated at the mRNA level in this model. (Figure 3D). As expected, the downregulation of PARP3 significantly impaired TGFβ-induced EMT. MCF10A-siPARP3 cells treated with TGFβ conserved a round-shaped epithelial phenotype (Figure 3A), maintained ZO1 staining of the cell borders (Figure 3B), showed no upregulation of Snail and no downregulation of E-cadherin (Figure 3C–3D). In contrast, at the mRNA level, *CDH1* was even upregulated in the PARP3-silenced cells (Figure 3D). Vimentin expression remained unchanged throughout time (Figure 3C–3D). We regularly noticed a decrease in the basal level of Snail upon PARP3 depletion, but without significant consequence on the expression levels of E-cadherin or Vimentin. An impaired upregulation of Snail and Vimentin and an inefficient downregulation of E-cadherin was also observed in the breast cancer MCF7 cells upon PARP3 silencing (Supplementary Figure S3A).

The TGFβ-induced EMT is characterized by increased cell motility, a critical step in tumour progression. Given the importance of PARP3 in promoting TGFβ-induced EMT, we examined the effect of PARP3 silencing on the TGFβ-induced cell motility using a wound-healing assay (Figure 3E) (Supplementary Movie S1). While a treatment with TGFβ enhanced the motility of the MCF10A cells as detected by an increased speed and efficient wound closure, this increase was abrogated in PARP3 depleted cells suggesting that PARP3 mediates TGFβ-stimulated cell motility.

Chemoresistance is an other phenotype that is acquired by cells undergoing EMT [4, 35]. Both TGFβ and PARP3 have been shown to promote cell survival and NHEJ-mediated repair in response to genotoxic agents inducing double-strand breaks [12, 29, 30, 33]. To test the importance of PARP3 in chemoresistance, we evaluated the sensitivity of TGFβ-treated MCF10A cells to etoposide, a potent inducer of double-strand breaks, after PARP3 depletion (Figure 3F). Relative to TGFβ untreated cells, MCF10A cultures treated with TGFβ became markedly less sensitive to etoposide indicating resistance to the DNA damage inflicted by etoposide. In contrast, PARP3-silenced MCF10A cells treated with TGFβ did not show this resistance arguing that the depletion of PARP3 abolished chemoresistance in cells undergoing TGFβ-mediated EMT. Similar results were obtained using bleomycin (Supplementary Figure S3B). These results suggested that the enhanced expression of PARP3 is required to confer chemoresistance in TGFβ-induced EMT.

### PARP3 promotes stem-like cell properties

**Figure 4:**
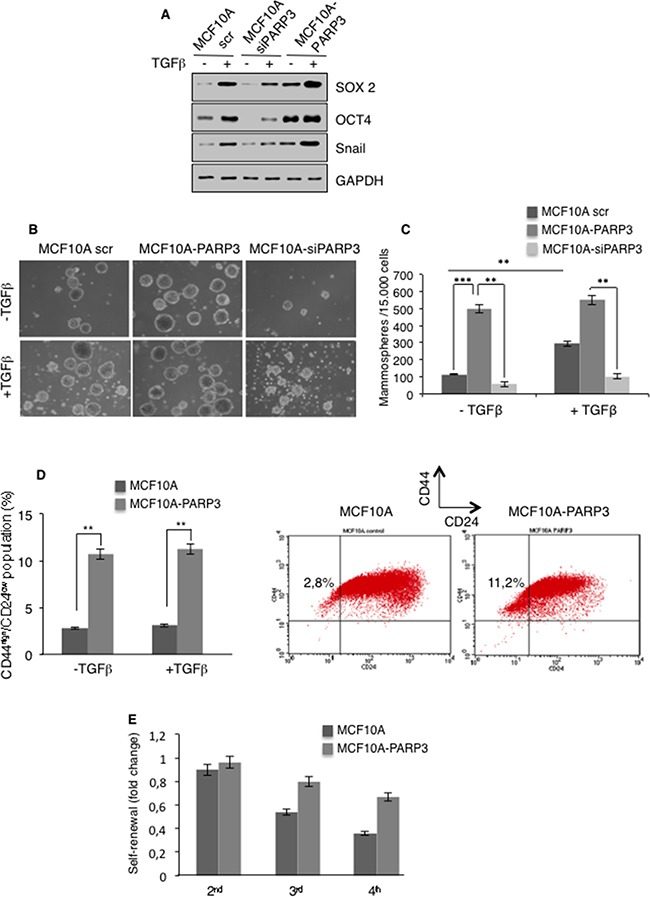
PARP3 promotes stem-like cell features in mammary epithelial cells **A.** Control (scr), PARP3-depleted (siPARP3) and PARP3 overexpressing (MCF10A-PARP3) MCF10A cells were mock-treated or incubated with TGFβ (10 ng/mL) for 72h. Expression of the stemness markers SOX2 and OCT4 and the EMT marker Snail were analysed by western blotting using the appropriate antibodies. GAPDH was used as a loading control. **B, C.** Control (scr), PARP3-depleted (siPARP3) and PARP3 overexpressing (MCF10A-PARP3) MCF10A cells were mock-treated or incubated with TGFβ (10 ng/mL) for 72h, grown as mammospheres for 9 days and quantified. Primary mammospheres were monitored under life-contrast microscopy (B) and counted for each condition (C). Histogram represents the average quantification (+/− s.d) of three independent experiments. **P<0,01, ***P<0,001. **D.** MCF10A control and MCF10A-PARP3 expressing cells were mock-treated or incubated with TGFβ (10 ng/mL) for 72h. The CD44^high^ /CD24^low^ sub-population was analysed by flow-cytometry. The percentage of the mean CD44^high^/CD24^low^ from two independent experiments is indicated. **E.** MCF10A control and MCF10A-PARP3 primary spheres grown in the absence of TGFβ were dissociated into single cells and seeded for subsequent sphere formation. Histogram depicts the quantification of serially passaged secondary (2^nd^), third (3^rd^) and fourth (4^th^) generation of mammospheres from three independent experiments.

There is evidence that EMT generates cells with stem-like characteristics [3–5]. In addition, TGFβ signaling has been implicated in stemness [36, 37]. On the basis of our results that PARP3 supports TGFβ-mediated EMT, we next investigated its participation in stem-like cell phenotypes. We examined the impact of PARP3 on the expression of representative stem cell markers in MCF10A cells in the absence or in the presence of TGFβ, using the above described PARP3 depletion or overexpression models (Figure 4A). In agreement with the litterature, TGFβ treatment resulted in the increase of the protein levels of the stemness markers SOX2 and OCT4 [37]. Compared to MCF10A cells, MCF10A-siPARP3 cells revealed significantly decreased basal and TGFβ-induced levels of OCT4 and the TGFβ-induced level of SOX2, while PARP3 overexpression in the MCF10A-PARP3 cells clearly enhanced the basal level of both markers and to a lesser extent their TGFβ-induced levels. Corroborating the role of PARP3 in TGFβ-induced EMT, the absence of PARP3 impaired the upregulation of Snail while its overexpression in the MCF10A-PARP3 clearly increased its basal and TGFβ-induced levels. Similar results were obtained using the breast cancer MCF7 cells (Supplementary Figure S4A). To further study the importance of PARP3 in breast stem cell activity, we used the tumor spheroids forming assay to evaluate the capacity for mammosphere formation in non-adherent serum-free conditions [38] (Figure 4B, 4C). MCF10A-PARP3 cells revealed a marked increase in the number of primary mammospheres compared to the MCF10A control cells independently of TGFβ. In turn, MCF10A-siPARP3 showed a decrease in the number and size of primary mammospheres with and without TGFβ. Similar results were observed in the MCF7 cells (Supplementary Figure S4B, S4C). Thus arguing that PARP3 is involved in primary spheroid bodies formation of mammary epithelial and breast cancer cells. Next, we analyzed PARP3-overexpressing MCF10A cells for the presence of tumor initiating CD44^high^/CD24^low^ population characteristic for the mammary and breast cancer stem cells (Figure 4D). Flow cytometric analysis revealed a significant 4-fold increase in the proportion of CD44^high^/CD24^low^ cells in the MCF10A-PARP3 versus the MCF10A control cells again independently of TGFβ. To explore the contribution of PARP3 in stem self-renewal, we examined the capacity of the MCF10A control and MCF10A-PARP3 primary spheres to generate secondary, third and fourth generation of mammospheres in serial passages (Figure 4E). MCF10A-PARP3 displayed an increased capacity to self-renew throughout passages compared to the MCF10A control cells. Similar results were observed in the MCF7 cells (Supplementary Figure S4D). Furthermore, throughout the serial passages of mammospheres, the protein level of the stemness marker OCT4 remained higher in the MCF7-PARP3 then in the MCF7 control cells (Supplementary Figure S4E). These results indicated that PARP3 helps to maintain stem cell renewal capacity in mammary epithelial and breast cancer cells.

Collectively, these data revealed that PARP3 endows human mammary epithelial cells and breast cancer cells with stem cell properties.

### PARP3 enhances TGFβ-driven EMT and stemness by stimulating the expression of the transglutaminase TG2

**Figure 5:**
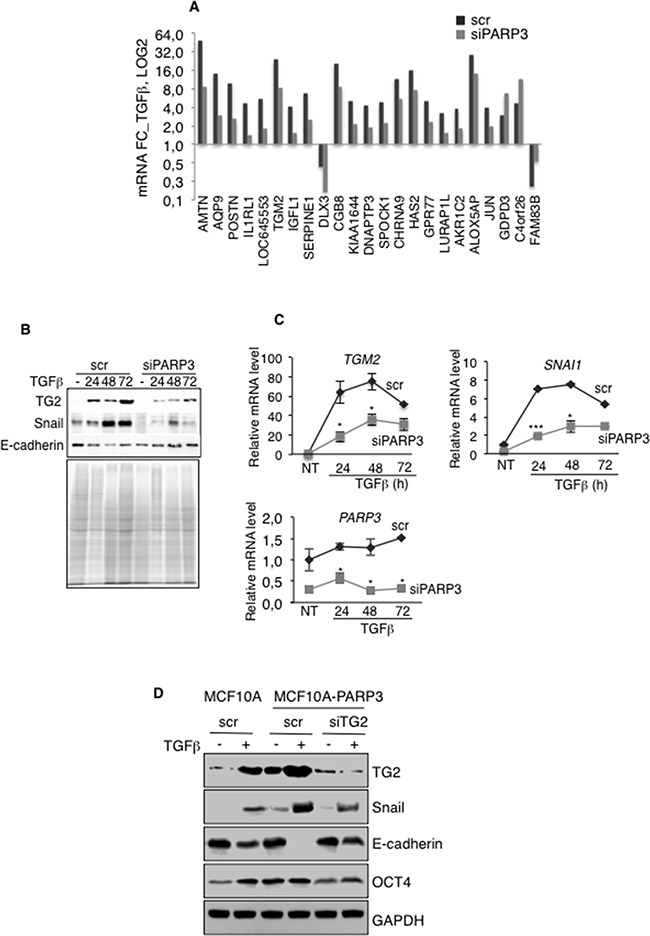
PARP3 promotes TGFβ-driven EMT via the regulation of the transglutaminase TG2 Control (scr) and PARP3-depleted (siPARP3) MCF10A cells were mock-treated or incubated with TGFβ for 48h and processed in triplicates for microarray analysis. **A.** Of the 538 TGFβ responsive genes, 23 genes were specifically and significantly deregulated in the PARP3 silenced versus the control MCF10A cells (fold change > 2 or < 0,5 and p value < 0,001). Histogram depicts the fold change of the 23 selected TGFβ-responsive genes. **B.** Time course protein expression levels of TG2, Snail and E-cadherin in control (scr) and PARP3 silenced (siPARP3) MCF10A cells either mock-treated or treated with TGFβ (2 ng/mL) for the indicated time points. Coomassie staining of the protein extracts was used as loading control. **C.** Time course RT-qPCR analysis of *TGM2*, *SNAI1* and *PARP3* mRNA levels in the control (scr) and PARP3 silenced (siPARP3) MCF10A cells left untreated or treated with TGFβ (2 ng/mL) for the indicated time points. *UBC* mRNA was used for normalization. Experiments were performed >3 times giving similar results. Error bars represent the mean (+/− s.d) of triplicates. Statistics were calculated on the differential expression between siPARP3 relative to scr. *P<0,05, ***P<0,001. **D.** MCF10A or MCF10A-PARP3 cells were transfected with either control siRNA (scr) or siTG2 to induce TG2 depletion for 48h and mock-treated or incubated with TGFβ (2 ng/mL) for an additional 72h. Expression of the EMT markers TG2, Snail, E-cadherin and the stemness marker OCT4 were analysed by western blotting using the appropriate antibodies. GAPDH was used as a loading control.

To explore the underlying mechanism of PARP3 functions in TGFβ-mediated EMT, we analysed the transcriptome changes in MCF10A cells upon PARP3 depletion and TGFβ treatment in MCF10A cells using a microarray approach (Figure 5A). From the 538 coding genes responding to TGFβ in our experimental conditions, we identified a handful of genes that were differentially deregulated in the PARP3-silenced MCF10A cells compared to the control MCF10A cells (23 genes, 0,5≥ FC^TGFβ^ ≥ 2, p value < 0,001). Among them, we focused our interest on the tissue transglutaminase TG2 that in a similar manner as PARP3 was documented to have prominent roles in TGFβ-induced EMT, the acquisition of stem cell features and chemoresistance [19, 39]. These similarities evoked a causal link between PARP3 and TG2.

We first validated the differential expression of TG2 in MCF10A and MCF10A-siPARP3 cells in response to TGFβ by a time course RT-qPCR and western blot analysis (Figure 5B, 5C). Consistent with efficient EMT in MCF10A cells, TG2 and Snail markers were up-regulated while E-cadherin was down-regulated in response to TGFβ at both mRNA and protein levels. As expected, deregulation of these EMT markers was significantly perturbed in MCF10A-siPARP3 cells, TG2 and Snail levels remained low and E-cadherin level high in agreement with impaired EMT as stated above (Figure 3). Similar results were obtained in the breast cancer MCF7 cells (Supplementary Figure S3A). Given that TG2 is required for the expression of Snail during EMT [16], these data suggested that PARP3 stimulates a TG2-Snail-E-cadherin axis to support TGFβ-dependent EMT.

To verify this hypothesis, we examined the impact of TG2 on Snail up-regulation and consequent repression of E-cadherin stimulated by TGFβ and PARP3 overexpression in MCF10A cells (Figure 5D). Compared to the MCF10A control cells with low basal PARP3, PARP3 overexpression in MCF10A-PARP3 cells increased the TGFβ-induced expression of TG2 and Snail and consequently decreased E-cadherin. Remarkably, these effects were significantly diminished upon TG2 depletion using siRNA. Thus confirming that in response to TGFβ, PARP3 promotes a TG2-Snail-E-cadherin axis to induce EMT. Similarly, the PARP3-stimulated expression of the stemness marker OCT4 detected in the absence or presence of TGFβ in MCF10A-PARP3 cells was diminished upon TG2 depletion indicating that the genesis of stem cells is also at least partly mediated by a PARP3-TG2 pathway.

### PARP3 responds to TGFβ-induced ROS to promote the TG2-Snail-E-cadherin axis during EMT

**Figure 6:**
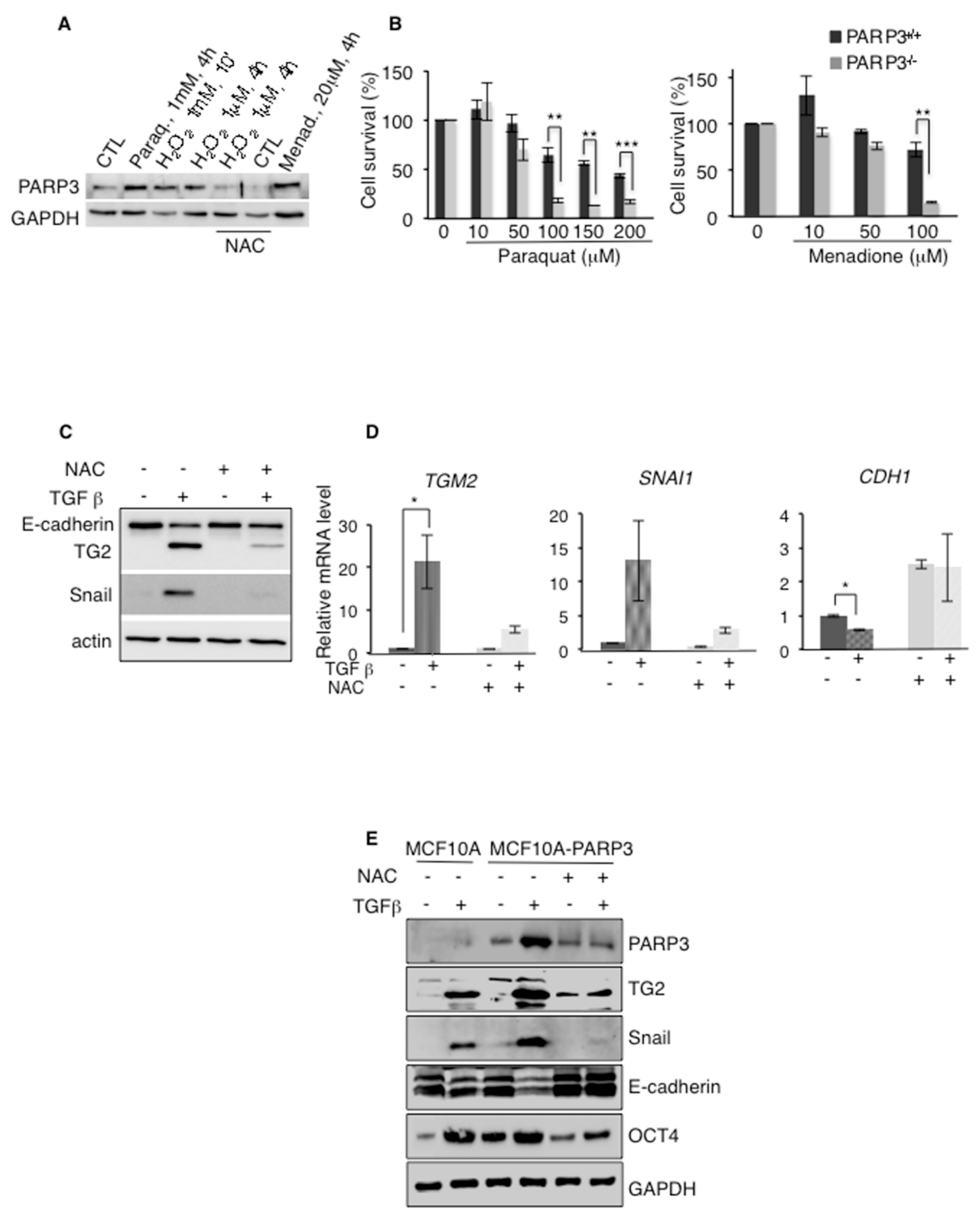
PARP3 stimulates a TG2-Snail-E-cadherin axis in response to TGFβ-driven ROS production **A, B.** PARP3 responds to and promotes survival upon the induction of oxidative damage. (A) Protein level of PARP3 upon exposure of MCF10A-PARP3 cells to H_2_0_2_, Paraquat or Menadione-induced oxidative stress. GAPDH was used as a loading control. When indicated, cells were pre-treated with the ROS scavenger N-acetyl cysteine (NAC) (10 mM, 2h) and NAC was maintained throughout. (B) Dose response clonogenic survival curves of Parp3^+/+^ and Parp3^−/−^ immortalised MEFs either mock-treated or exposed to increasing concentrations of Paraquat (left) and Menadione (right). Exponentially growing MEFs were treated with Paraquat or Menadione at the indicated concentrations for 19h, rinsed twice with PBS and seeded for clonogenicity. Experiments were performed 3 times giving similar results. Mean values of triplicates (+/− s.d) are shown. **P<0,01, ***P<0,001. **C, D.** TGFβ-mediated ROS production is involved in the expression of the EMT markers TG2, Snail and E-cadherin. MCF10A cells were mock-treated or incubated with TGFβ for 48h in the absence or in the presence of NAC. In the latter condition, cells were pre-treated with NAC (10 mM, 2h) and NAC was maintained throughout. TG2, Snail and E-cadherin expression levels were analysed (C) by Western blotting using the appropriate antibodies. Actin was used as loading control. (D) and by RT-qPCR. *UBC* mRNA was used for normalization. Error bars represent the mean (+/− s.d) of triplicates. *P<0,05 **E.** TGFβ-mediated ROS production is involved in the increase of PARP3 protein level and PARP3 overexpression results in increased protein levels of the EMT markers TG2, Snail, E-cadherin and of the stemness marker OCT4 in a TGFβ and ROS dependent manner. MCF10A and MCF10A-PARP3 cells were mock-treated or exposed to TGFβ for 48h in the absence or in the presence of NAC. In the latter condition, cells were pre-treated with NAC (10 mM, 2h) and NAC was maintained throughout. EMT and stemness markers were analysed by western blotting using the specific antibodies. GAPDH was used as a loading control.

Accumulating evidence suggests that ROS play important roles in TGFβ-induced EMT [10]. In pursuit of the mechanism by which PARP3 regulates the TG2-Snail-E-cadherin axis and promotes EMT, we decided to investigate the importance of ROS in this pathway (Figure 6). We first asked whether PARP3 is involved in cell response to oxidative damage using the MCF10A-PARP3 cells. Western blotting revealed an increase in the protein level of PARP3 upon treatment with the ROS inducing agents H_2_0_2,_ paraquat and menadione when compared to the untreated cells. PARP3 upregulation upon H_2_0_2_ treatment was abolished in the presence of the ROS scavenger, N-acetyl cysteine (NAC) (Figure 6A). We then examined the sensitivity of the Parp3^+/+^ and Parp3^−/−^ mouse embryonic fibroblasts to paraquat and menadione by clonogenic survival assays. The absence of Parp3 rendered cells hypersensitive to both drugs (Figure 6B). These findings uncovered an important role of PARP3 in cellular response to the generation of ROS.

Next we tested whether TGFβ-induced ROS production contributed to the enhanced expression of TG2 and Snail, or the repression of E-cadherin in MCF10A cells. The treatment with NAC notably reduced the TGFβ-dependent expression of TG2 and Snail and the concomittant repression of E-cadherin at both mRNA and protein levels, indicating that the TGFβ-induced expression of EMT markers is ROS dependent (Figure 6C, 6D). Similarly, the TGFβ-dependent upregulation of PARP3 in the MCF10A-PARP3 was notably reduced in the presence of NAC (Figure 6E). The above findings prompted us to interrogate whether PARP3 mediates the TGFβ and ROS dependent activation of the TG2-Snail-E-adherin axis and the expression of the stemness marker OCT4 (Figure 6E). As described above (Figure 5), compared to the MCF10A control cells displaying low levels of endogenous PARP3, PARP3 overexpression in MCF10A-PARP3 enhanced the TGFβ-stimulated expression of TG2 and Snail and further repressed E-cadherin. These effects were significantly diminished in the presence of NAC demonstrating the importance of ROS. Similarly, the PARP3-stimulated expression of the stemness marker OCT4 detected in the absence or presence of TGFβ was abrogated in the presence of NAC. Comparable results were obtained in the MCF7 cells (Supplementary Figure S5).

Collectively, these findings identified PARP3 as a TGFβ and ROS responsive protein and demonstrated that PARP3 supports TGFβ-dependent EMT traits in a ROS dependent manner by stimulating a TG2-Snail-E-cadherin axis.

## DISCUSSION

Based on our initial observation that PARP3 levels are generally higher in the mesenchymal-like basal B breast cancer cell lines that display EMT properties and correlate with the EMT score, we aimed to decipher the contribution of PARP3 in this process. We report that PARP3 serves TGFβ-dependent EMT and stemness by stimulating a TG2-Snail-E-cadherin axis in a manner involving its response to TGFβ-induced ROS.

This mode of regulation reveals a novel function of PARP3 in cellular response to ROS, in addition to its well characterized implication in double-strand break repair [29, 30, 33]. The fact that PARP3 is upregulated and responds to ROS produced from both exogenous (genotoxic agents) and endogenous (TGFβ) sources makes it a major driver of the ROS response that is likely to function in various other physiological and pathophysiological events. One interesting example is suggested by the prominent role of PARP3 in neural crest cell differentiation in the zebrafish [32], a process in which the generation of ROS by the NADPH oxidase NOX4 was documented to be required for efficient differentiation [40]. Whether PARP3 might be involved in the base excision repair (BER) pathway that processes oxidative lesions is undetermined, but consistent with its interaction with the BER proteins PARP1 and DNA ligase III and the recent biochemical demonstration that PARP3 can be selectively activated by 5′ phosphorylated DNA breaks, defined as prevalent intermediates of the BER pathway [41, 42].

The causal relationship between PARP3 and TG2 to regulate the EMT master regulator Snail is grounded by the considerable functional similarities between these proteins : (i) they promote mesenchymal properties, cell migration, wound healing [39, 43, 44]; (ii) their expression is induced by genotoxic stress and they prime a drug resistance phenotype [29, 45, 46]; (iii) they confer cancer stem-like cell traits [18, 44]. The microarray and RT-qPCR analyses suggest that PARP3 controls the transcriptional expression of *TGM2*. We have yet to elucidate the mechanism involved. An interesting hypothesis originates from the existence of a TG2/NF-κB self-reinforcing signaling loop identified in breast cancer cells, and that was proposed to influence EMT and produce a drug-resistant phenotype [47]. TG2 was found to activate NF-κB and was reciprocally identified as a direct transcriptional target of NF-κB [47, 48]. Most notably, it was proposed that in response to cellular stressors such as oxidative stress, an ATM-dependent activation of the canonical NFκB pathway drives heightened expression of TG2 [45]. In view of the role of PARP3 in the activation of ATM [31] and the PARP3 response to ROS identified here, one possible mechanism would be that PARP3 guides an ATM-NF-κB-TG2-Snail route to boost TGFβ-induced EMT and cause drug-resistance. A fuller appreciation of this mechanism will require a more detailed study on how PARP3 responds to oxidative stress and whether such a redox sensor activity may, either directly or indirectly engage the ATM-NF-κB couple.

The fact that PARP3 contributes to the drug resistance phenotype mediated by TGFβ is substantiated by the recent findings that both PARP3 and TGFβ accelerate NHEJ repair activity in two similar ways, by facilitating the retention of DNA ligase IV at the sites of DNA damage and by promoting ATM activation [12, 13, 31, 33]. Although the mechanism involved remains to be determined, it is reasonable to speculate that the upregulation of PARP3 represents one possible way by which TGFβ facilitates NHEJ.

The other outstanding result of this study is the marked role of PARP3 in the genesis and function of mammary and breast cancer stem cells illustrated by its contribution in mammosphere formation, the expression of the stemness markers OCT4 and SOX2, and the frequency of the tumor initiating CD44^high^/CD24^low^ population. The puzzling observation is the strict TGFβ-dependence of PARP3 to regulate the mesenchymal properties while the importance of TGFβ for the modulation of stem-like traits is less pronounced. This might be the result of a broader role of PARP3 in stem cell development. PARP3 might not only regulate TGFβ-dependent EMT for the emergence of stem cells, but also other specific stem cell regulators that play causal roles in stemness. The importance of ROS in this function is suggested by the finding that PARP3 regulates the expression of OCT4 in a ROS-dependent manner. In view of the critical importance of cancer stem cells in tumor dissemination, the observation that PARP3 promotes stem-like cell characteristics supports our initial observation that invasive basal-like cancer cell lines express higher basal levels of PARP3. An other interesting hypothesis to consider in forthcoming studies is the link between PARP3 and the Polycomb Repressive Complex (PRC2) for the regulation of EMT and stemness. Indeed, PARP3 associates with components of the PRC2 complex, both proteins share the regulation of overlapping neural developmental genes and the role of PRC2 in the epigenetic control of TGFβ-induced EMT and stem cell development has been documented in various normal and disease contexts [32, 42, 49, 50].

In conclusion, our data provide evidence that PARP3 drives TGFβ-mediated EMT and stem cell development in normal mammary and breast cancer cells and that these activities involve a PARP3 response to the generation of ROS. In the cancer field, EMT markers and mediators have been particularly associated with basal invasive breast cancer subtypes with often a poor clinical outcome. The identification of EMT drivers as specific therapeutic targets represents a continuous challenge. Similarly, given the importance of cancer stem cells in drug resistance, tumor initiation and tumor recurrence, clarifying the mechanisms involved has important therapeutic implications. Consequently, our findings support the idea that targeting PARP3 may have a clear potential benefit to restrain cancer related TGFβ-promoted EMT and may be used as an advantage to target cancer stem cells. The current active tracking in the development of potent cell-permeant PARP3 inhibitors will be valuable in exploring these strategies [51–53].

## MATERIALS AND METHODS

### Cell culture

MCF10A, BT20, T47D, ZR751 and HepG2 were obtained from the Cell Culture facility (IGBMC, Illkirch). MDA-MB468, HCC1937, MDA-MB231, MDA-MB436, Hs578T and HCC38 were obtained from the American Type Culture Collection (ATCC). MCF10A were maintained in DMEM:F12_HAM (1 :1) medium supplemented with 5% horse serum, 10 μg/mL Insulin, 20 ng/mL hEGF, 100 ng/mL Cholera Toxin, 0,5 mg/mL hydrocortisone, 40 μg/mL gentamicin. T47D, MDA-MB468, HCC1937, MDA-MB436, MDA-MB231 were maintained in RPMI medium supplemented with 10% FCS and 1% gentamicin. A549 and Hs578T were maintained in DMEM-1g/L D-glucose medium supplemented with 10% FCS and 1% gentamicin. All cell lines were maintained at 37°C in a humidified 5% CO2 atmosphere. Parp3^+/+^ and Parp3^−/−^ immortalised MEFs were described [29].

MCF10A-PARP3 were generated by lentiviral infection using the custom-made lentiviral expression vector pLenti-III-CMV-hPARP3 (ABM, LV018933) and selection in puromycin (1 μg/mL for MCF10A).

Gene-specific siRNA for PARP3 (ON_TARGET plus human PARP3 J-009297), TG2 (ON_TARGET plus smart pool, L-004971) and negative control siRNA scr (scrambled) were obtained from Dharmacon (Thermo Scientific). Cells were transfected with 50 nM siRNA using JetPrime (PolyPlus Transfection) according to the manufacturer's instructions and processed 40h later.

TGFβ2, Paraquat and Menadione were purchased from Sigma-Aldrich. Etoposide was purchased from Mylan Laboratories Inc. N-Acetyl-Cystein (NAC) was purchased from Santa Cruz Biotech.

### Microarray and RT-qPCR analysis

Total RNA was extracted from cells using the RNAeasy kit (Qiagen) according to the manufacturer's protocol.

For RT-qPCR, DNase-treated RNA was processed for reverse transcription using the Maxima Reverse Transcriptase (Thermo Scientific) according to the manufacturer's instructions. Real time PCR was performed using the QuantiTect SYBR Green PCR kit following the manufacturer's instructions (Qiagen) combined with the Applied Biosystems StepOne (Life technologies) detection system. The PCR products were analysed with the StepOne Software. The quantity of PCR products was estimated by the relative standard curve method and the ΔΔCt method. All samples were analyzed in triplicates and normalized using the *GAPDH or TBP* housekeeping genes as indicated. The primer sequences used for qPCR are listed in Supplementary Table S2.

For microarray analysis, biotinylated single strand cDNA targets were prepared, starting from 150 ng of total RNA, using the Ambion WT Expression Kit (4411974) and the Affymetrix GeneChip® WT Terminal Labeling Kit (900671) according to Affymetrix recommendations. Following fragmentation and end-labeling, 3 μg of cDNAs were hybridized for 16 hours at 45°C on *GeneChip® Human Gene 2.0 ST arrays* (Affymetrix) interrogating over 40 0000 RefSeq transcripts and ~ 11000 LncRNAs represented by approximately 27 probes spread across the full length of the transcript. The chips were washed and stained in the GeneChip® Fluidics Station 450 (Affymetrix) and scanned with the GeneChip® Scanner 3000 7G (Affymetrix) at a resolution of 0.7 μm. Raw data (.CEL Intensity files) were extracted from the scanned images using the Affymetrix GeneChip® Command Console (AGCC) version 4.0. CEL files were further processed with Affymetrix Expression Console software version 1.3.1 to calculate probe set signal intensities using Robust Multi-array Average (RMA) algorithms with default settings. Differentially expressed genes were selected using the fold change based method [54].

### Western blot analysis

For PARP3 expression analysis in HBC cell lines (Figure 1B), cells were collected and lysed by incubation in ice for 10 minutes in RIPA-like buffer (50mM Tris pH 8, 1% triton, 0,5% sodium deoxycholate, 150 mM NaCl, 1mM EDTA, 50 mM NaF, 20 mM sodium pyrophosphate pH 7,2, 1 mM sodium orthotovanadate, 1 mM Pefabloc, PIC). After centrifugation at 10000 rpm at 4°C for 20 min, cleared suspension was quantified by Bradford protein assay. Equivalent amounts of proteins were next analysed by 10% SDS-PAGE and immunoblotting using the appropriate antibodies (Listed in Supplementary Table S1). Protein bands were visualized using ECL-PLUS detection system (Amersham Biosciences) and the images were captured using the Image Quant LAS 4000 imaging system (GE Healthcare Life Science).

For all other analysis of protein levels, cells were washed twice in 1X PBS containing 0,5mM Pefabloc, collected by scraping in 4X Lysis Buffer (250mM Tris-HCl pH 6,8, Glycerol 40%, SDS 8%, DTT 50 mM, Blue Bromophenol 0,01%, Na_3_VO_4_ 1mM, NaF 50mM, Pefabloc 0,5mM added before use) and sonicated using Bioruptor UCD-300 system. Normalization of the samples was performed by running a 10% SDS-PAGE and in-gel quantification of total protein stained with Coomassie using the Odyssey Infrared Imaging System (*LI-COR* Biosciences) and the analysis software Image J. Equivalent amounts of proteins were next analysed by 10% SDS-PAGE and immunoblotting using the appropriate antibodies (Listed in Supplementary Table S1). Protein bands were visualized as mentionned above.

### Immunofluorescence microscopy

Immunofluorescence analysis was performed as described [55]. Slides were mounted in Mowiol. Images were captured using a Leica microscope (Leica Microsystems) equiped with an ORCA-ER chilled CCD camera (Hammamatsu) and the capture software Openlab (Improvision).

### Colony-forming assay

Cells (2.10^3^) were seeded in 100-mm culture dishes in triplicate. Seven days later, cells were fixed for 30 min in formaldehyde (4%), stained with crystal violet (0,1%) and colonies were scored.

### Mammosphere culture and self-renewal capacity

Mammospheres were prepared as described [38]. Briefly, single cell suspensions (15.10^3^) were plated in poly-2-hydroxyethylmethacrylate-treated (pHEMA, Sigma, St. Louis, MO) 6-well plates in serum-free DMEM-F12 medium supplemented with hEGF 20 ng/ml, bFGF 20 ng/ml, L-Glutamine 1%, B27 supplement 2%. This medium was semi-solidified by the addition of 0.5% Methylcellulose (HSC001 RD system). After 9 days in culture, mammospheres were counted and images were captured in a Leica DFC 425C Digital Microscope Camera. To evaluate the self-renewal capacity, primary spheres were collected by centrifugation (1000 rpm 5 minutes) and dissociated enzymatically (5 min in 1:1 trypsin/DMEM solution at 37°C) and mechanistically by pipetting. Single cells (1.10^3^) were re-plated in pHEMA 6-well plates for 10 days for subsequent mammosphere formation.

Identification of CD44^High^/CD24^Low^ population was performed using monoclonal anti-CD44-FITC (clone G44-26) and anti-CD24-PE (clone ML5) antibodies (BD Biosciences). Cells were labelled and CD44/CD24 markers were analysed using a FACSCalibur Flow Cytometer and the Cell Quest Software (Becton Dickinson).

### Scratch wound assays

A linear wound was generated using a sterile 200μl pipette tip and cell monolayers were placed on an environment-controlled Confocal Leica IRBE 2 microscope equipped with a Leica DC 350 FX camera and the Imaging capture software FW 4000, and imaged every 20 mn for 48h using a 10X bright-field objective.

### Statistical analysis

The data are shown as +/− s.d. P-values (*) were determined using Student's t-test. For correlation studies, statistical significance was calculated by Pearson's correlation.

## SUPPLEMENTARY MATERIALS AND METHODS


