# Supplementary material for: PARP3 controls TGFβ and ROS driven epithelial-to-mesenchymal transition and stemness by stimulating a TG2-Snail-E-cadherin axis
Source: Oncotarget. 2016 Aug 26;7(39):64109–23. doi: 10.18632/oncotarget.11627 (PMC5325429; doi:10.18632/oncotarget.11627)
Supplement: Supplementary file 1 [file oncotarget-07-64109-s001.pdf]

PARP3 controls TGFβ and ROS driven epithelial-to-mesenchymal transition and stemness by stimulating a TG2-Snail-E-cadherin axis

SUPPLEMENTARY MATERIALS AND METHODS

Generation of MCF7-PARP3 cell lines

The human breast cancer cell line MCF7 was obtained from the Cell Culture facility (IGBMC, Illkirch). MCF7 were cultivated in DMEM-1g/l D-glucose medium supplemented with 10% FCS and 1% gentamicin and maintained at 37°C in a humidified 5% CO_2 atmosphere. MCF7-PARP3 cell lines were generated by lentiviral infection using the custom-made lentiviral expression vector pLenti-III-CMV-hPARP3 (ABM, LV018933) and selection in puromycin (2 µg/mL).

SUPPLEMENTARY TABLES AND FIGURES

Supplementary Table S1: List of antibodies used in the study

Name	Company	Concentration
Rabbit anti-PARP3, 4698	Home made [1]	WB, 1 :10000
Mouse anti-PARP1, EGT69	Home made	WB, 1 :10000
Mouse anti-E-cadherin, 61018	BD Biosciences	WB, 1 :5000
Mouse anti-Vimentin, RV202	Santa Cruz Biotech	WB, 1 :1000
rabbit anti-GAPDH, G9545	Sigma-Aldrich	WB, 1 : 10000
goat anti-E-cadherin, AF648	RD System	IF, 1 :100
mouse anti-ZO1, 339100	Invitrogen	IF, 1 :500
rabbit anti-Snail, C15D3	Cell Signaling Technology	WB, 1 :1000
rabbit anti-OCT4, ab80700	Abcam	WB, 1 :1000
rabbit anti-actin, A2066	Sigma-Aldrich	WB 1 :10000
mouse anti-SOX2, 245610	RD systems	WB : 1 :2000
rabbit anti-TG2, EP2957	Abcam	WB : 1 :10000
anti-CD44-FITC, clone G44-26	BD Biosciences	FACS, 1 :25
anti-CD24-PE, clone ML5	BD Biosciences	FACS, 1 :25
peroxidase conjugated goat anti-rabbit	GE Healthcare	WB: 1 :50000
peroxidase conjugated sheep anti-mouse	GE Healthcare	WB, 1 :30000
Alexa Fluor-488 goat anti-rabbit IgG	Molecular Probes	IF, 1 :1500
Alexa Fluor-568 goat anti-rabbit IgG	Molecular Probes	IF, 1 :1500

Supplementary Table S2: List of PCR primers used in the study

Gene	Séquences
PARP3	Fwd 5’-TGGCAAGGGCATCTACTTTG-3’<br>Rev 5’-TCCGTGTTGATATGGTGCTC-3’
VIM	Fwd 5’-GCAGGAGGAGATGCTTCAGA-3’<br>Rev 5’-ATTCCACTTTGCGTTCAAGG-3’
CDH1	Fwd 5’-TCCTGGCCTCAGAAGACAGA-3’<br>Rev 5’-GTGTTCACATCATCGTCCGC-3’
SNAI1	Fwd 5’-CTACAAGGCCATGTCCGGAC-3’<br>Rev 5’-GCCTGGCACTGGTACTTCTT-3’
TGM2	Fwd 5’-CACTTTGAGGCCCGCAACTA-3’<br>Rev 5’-TAGTGGAAAACGGGCCTTGG-3’
TBP	Fwd 5’-CAGCTTCGGAGAGTTCTGGG-3’<br>Rev 5’-TATATTCGGCGTTTCGGGCA-3’
UBC	Fwd 5’-ATTTGGGTCGCGGTTCTTG-3’<br>Rev 5’-TGCCTTGACATTCTCGATGGT-3’

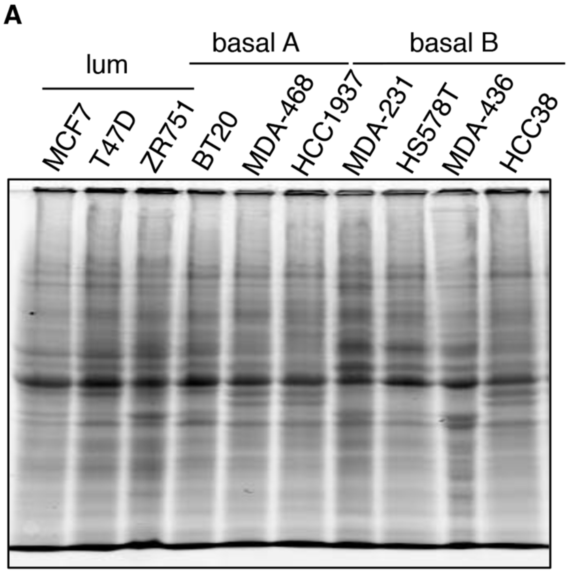

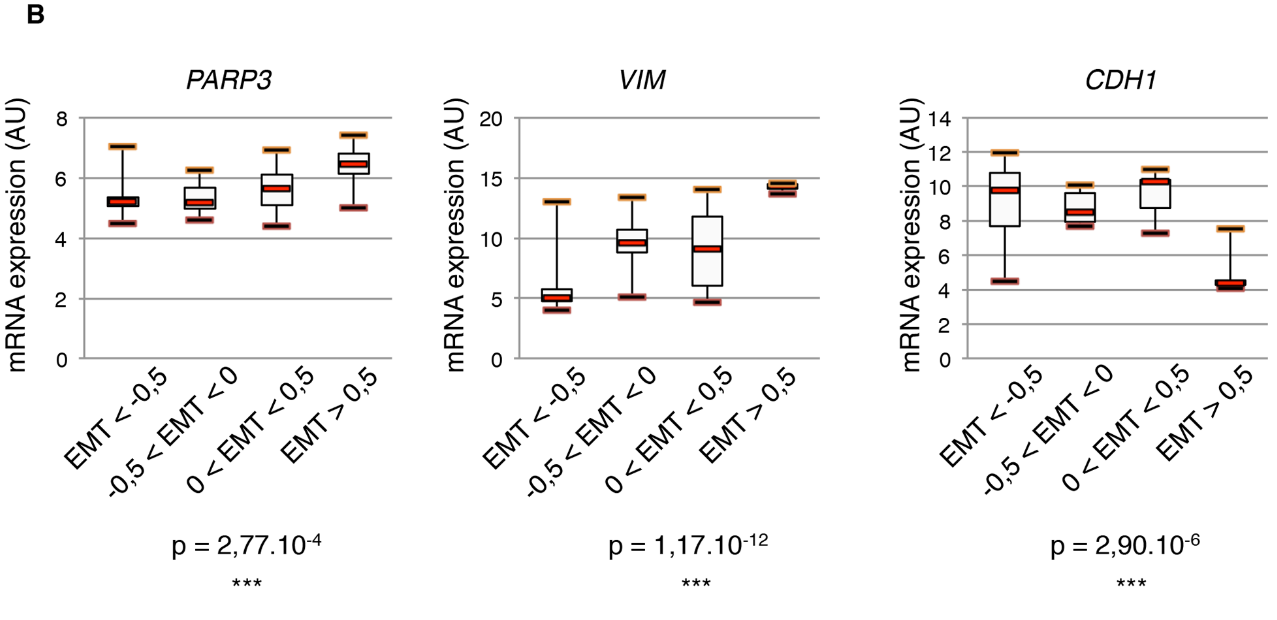

Supplementary Figure S1: A. Coomassie staining of the total protein extracts for the experiment shown in Figure 1B. **B.** Relative transcript abundance of *PARP3*, *VIM* and *CDH1* in human breast cancer cell lines according to the gene expression data set from the Cancer Cell line Encyclopedia (CCLE), correlated with the EMT score [2]. Breast cancer cell lines (n=44) are classified as the most epithelial-like (Epi) (score <-0.5) to two-intermediate state -0.5<EMT<0 and 0<EMT<0,5 and to the most mesenchymal-like (Mes) (score >0.5) according to the EMT signature defined in Tan et al [3]. P-value is calculated between the Epi (score <-0.5) and the Mes (score >0.5) population.

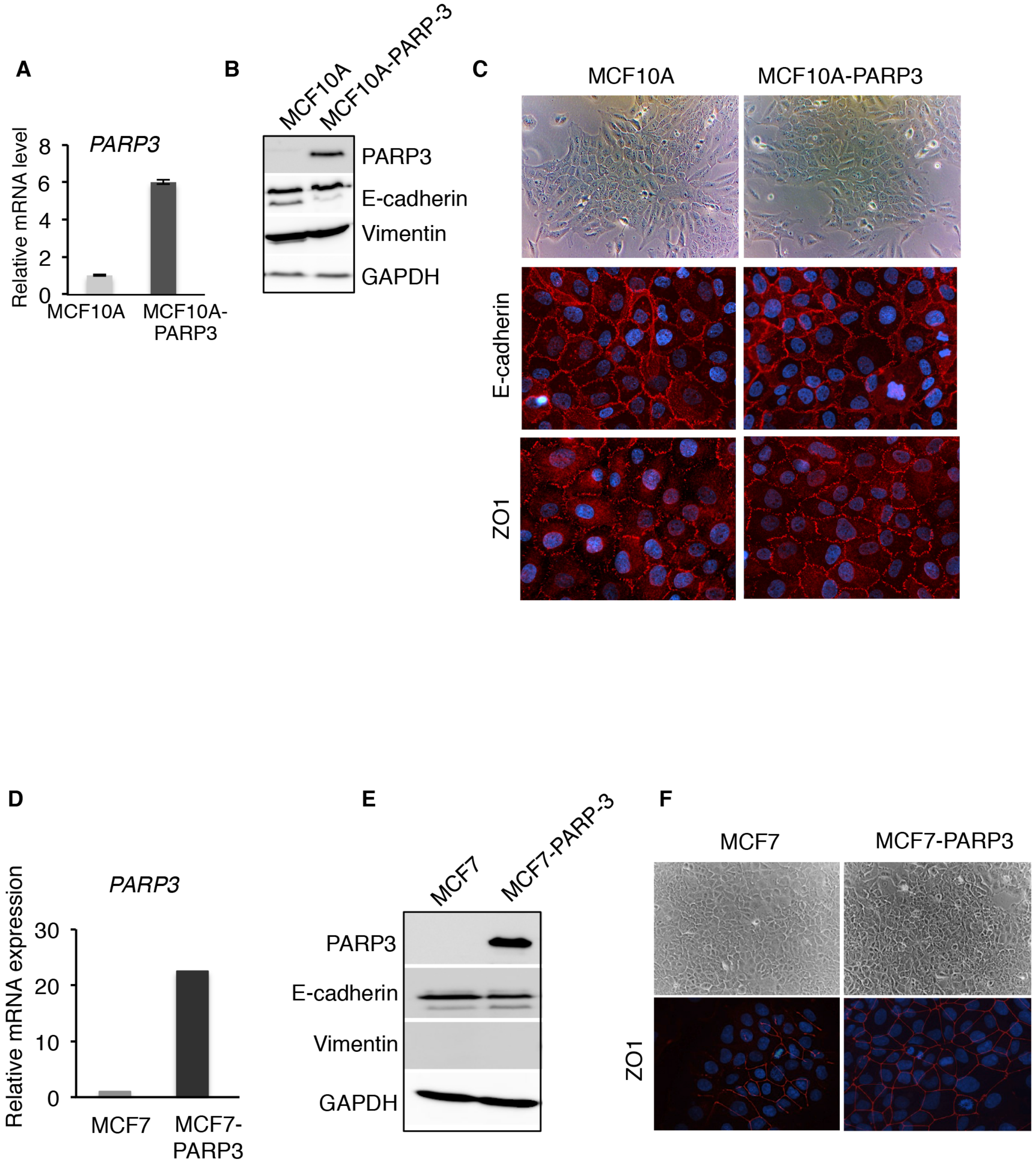

Supplementary Figure S2: Modulating PARP3 expression is not sufficient to trigger spontaneous EMT in mammary epithelial MCF10A and breast cancer MCF7 cells. A. MCF10A-PARP3 cells constitutively expressing PARP3 were generated by lentiviral infection using the custom made lentivirus pLenti-III-CMV-hPARP3 and confirmed by RT-qPCR for *PARP3* expression. **B.** The protein levels of PARP3 and EMT markers were analysed by western blotting using the specific antibodies. GAPDH was used as a loading control. **C.** Cells were monitored for morphological changes using phase contrast microscopy and by immunofluorescence for the EMT markers ZO1 (red) and E-cadherin (red). Nuclei were counterstained with DAPI (blue). Ectopic expression of PARP3 in MCF10A did not induce the loss of the junction proteins E-cadherin or ZO1 and no increase in the mesenchymal marker Vimentin. **D.** MCF7 constitutively expressing PARP3 were generated by lentiviral infection using a custom made lentiviral vector pLenti-III-CMV-hPARP3 and confirmed by RT-qPCR for PARP3 expression. **E.** The expression of PARP3, E-cadherin and Vimentin were analysed by western blotting using the specific antibodies. GAPDH was used as the loading control. Ectopic expression of PARP3 did not induce the loss of E-cadherin and no increase of Vimentin **F.** Cells were monitored for morphological changes using phase contrast microscopy and by immunofluorescence for the junction protein ZO1 (red). Nuclei were couterstained with DAPI (blue). PARP3-expressing MCF7 cells did not underwent striking morphological transformations and there was no dissolution of the ZO1-stained tight junctions.

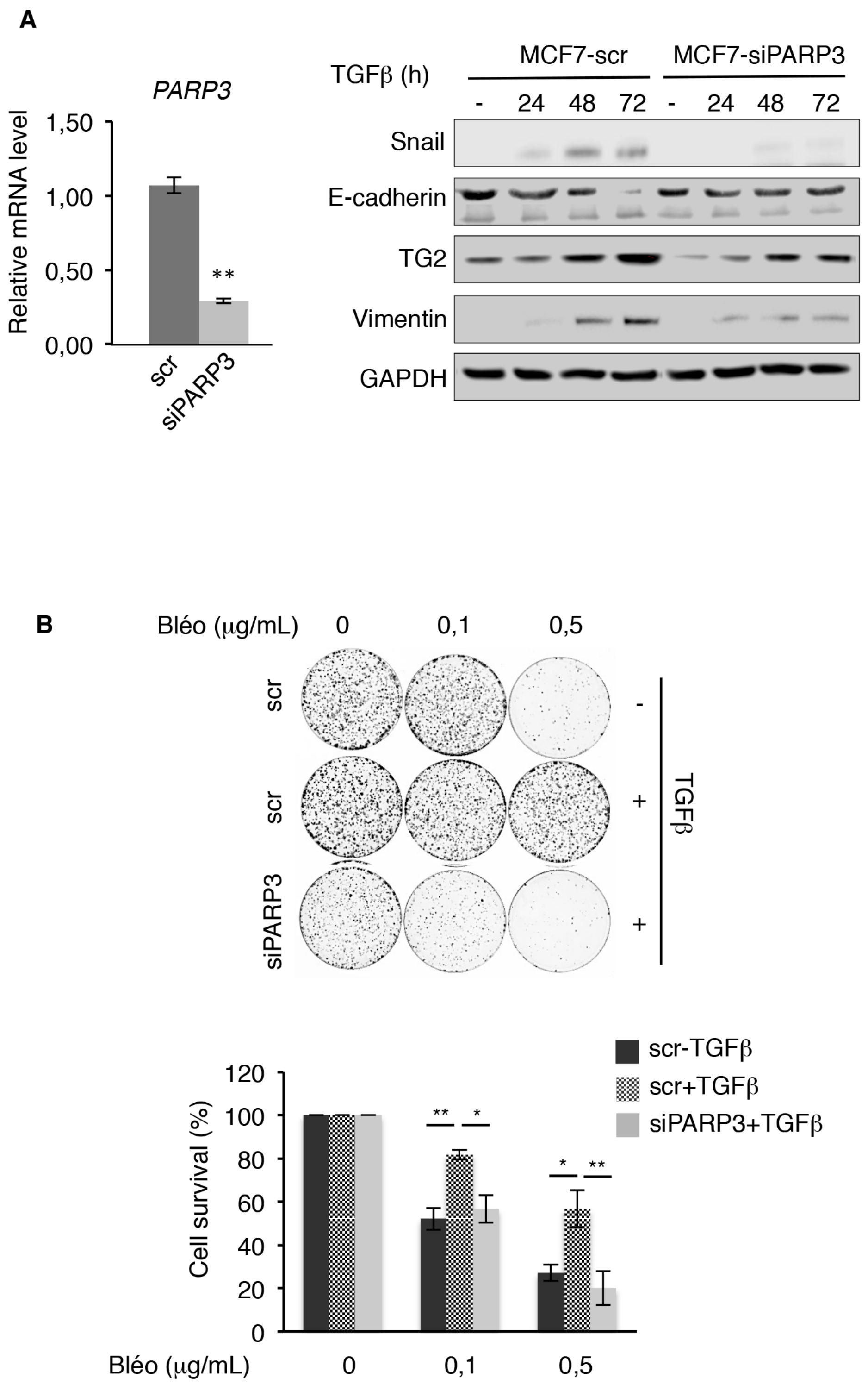

Supplementary Figure S3: PARP3 silencing impairs TGFβ-induced expression of EMT markers in MCF7 and TGFβ-mediated resistance to bleomycin in MCF10A. A. Control (scr) and PARP3 depleted (siPARP3) MCF7 cells were either mock-treated or incubated with TGFβ (2 ng/mL) for the indicated time points. EMT markers were analysed by western blotting using the appropriate antibodies. GAPDH was used as the loading control. **B.** TGFβ confers enhanced resistance to bleomycin that is reduced upon PARP3 silencing. Dose response clonogenic survival curves of control (scr) or PARP3 depleted (siPARP3) MCF10A cells mock-treated or treated with TGFβ (2 ng/mL) for 24h and exposed to increasing concentrations of bleomycin. Experiments were performed 3 times. Mean values of triplicates (+/- s.d) are shown. *P<0,05 ; ***P<0,01.

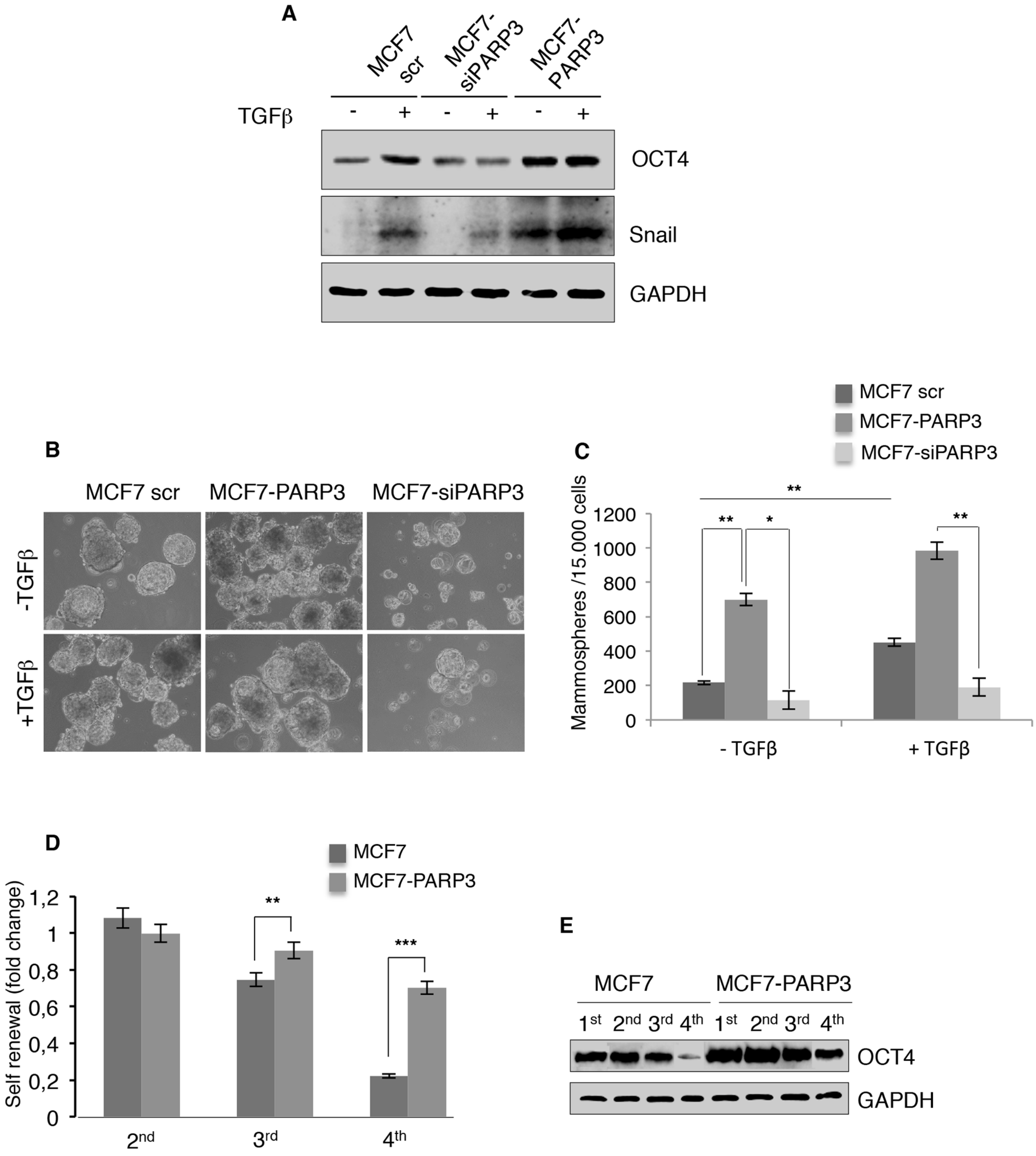

Supplementary Figure S4: PARP3 promotes stem-like cell properties in MCF7 cells. A. Control (scr), PARP3-depleted (siPARP3) and PARP3 overexpressing (MCF7-PARP3) MCF7 cells were mock-treated or incubated with TGFβ (10 ng/mL) for 72h. Expression of the stemness marker OCT4 and the EMT marker Snail were analysed by western blotting using the appropriate antibodies. The expression of GAPDH was used as a loading control. **B,C.** The ectopic expression of PARP3 in MCF7 increases the number of mammospheres in TGFβ treated (+TGFβ) and untreated (-TGFβ) cells while its depletion reduces the number of mammospheres in both conditions. Control (scr), PARP3-depleted (siPARP3) and PARP3 overexpressing (MCF7-PARP3) MCF7 cells were mock-treated or incubated with TGFβ for 72h, grown as mammospheres for 9 days and quantified. Primary mammospheres were monitored under life-contrast microscopy (B) and counted for each condition (C). Histogram represents the average quantification (+/- s.d) of three independent experiments. *P<0,05, **P<0,01. **D.** MCF7 control and MCF7-PARP3 primary spheres grown in the absence of TGFβ were dissociated into single cells and seeded for subsequent sphere formation. Histogram depicts the quantification of secondary (2nd), third (3rd) and fourth (4th) generation of mammospheres. Primary (1st), secondary (2nd), third (3rd) and fourth (4th) generation of MCF7 control and MCF7-PARP3 spheres were collected and analysed by western blotting for the protein level of the stemness marker OCT4. The expression of GAPDH was used as a loading control.

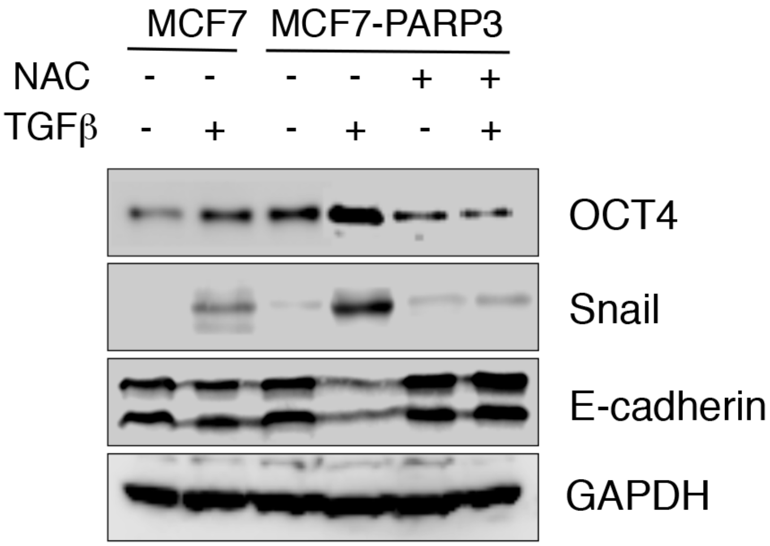

Supplementary Figure S5: PARP3 promotes the expression of OCT4 and Snail in MCF7 cells in a ROS-dependent manner. MCF7 and MCF7-PARP3 cells were mock-treated or exposed to TGFβ for 48h in the absence or in the presence of the ROS scavenger N-acetyl cysteine (NAC). The stemness marker OCT4 and the EMT markers Snail and E-cadherin were analysed by western blotting using the specific antibodies. GAPDH was used as a loading control. The ectopic expression of PARP3 upregulates the expression of the stem cell marker OCT4 in the absence and presence of TGFβ. This increase is lost in the presence of NAC revealing the importance of ROS. The expression of PARP3 upregulates the TGFβ-dependent increase of Snail and consequently downregulates the expression of the epithelial marker E-cadherin. These changes are lost in the presence of NAC revealing the contribution of ROS.

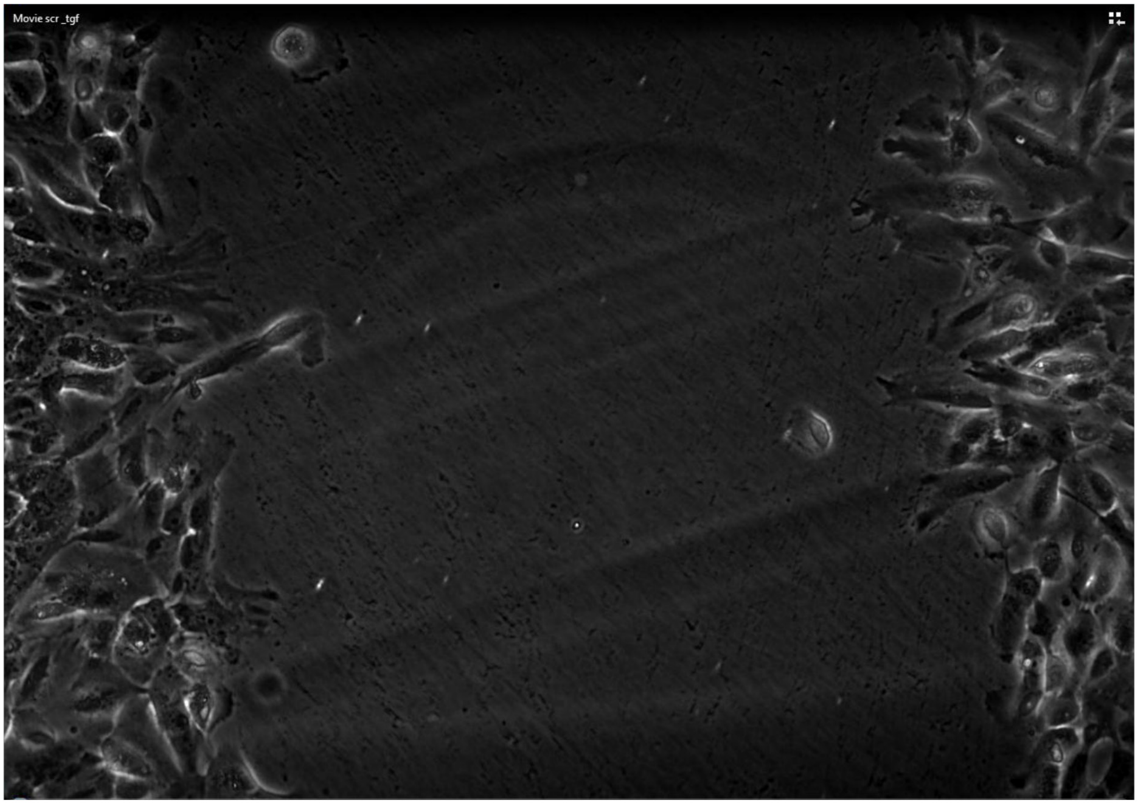

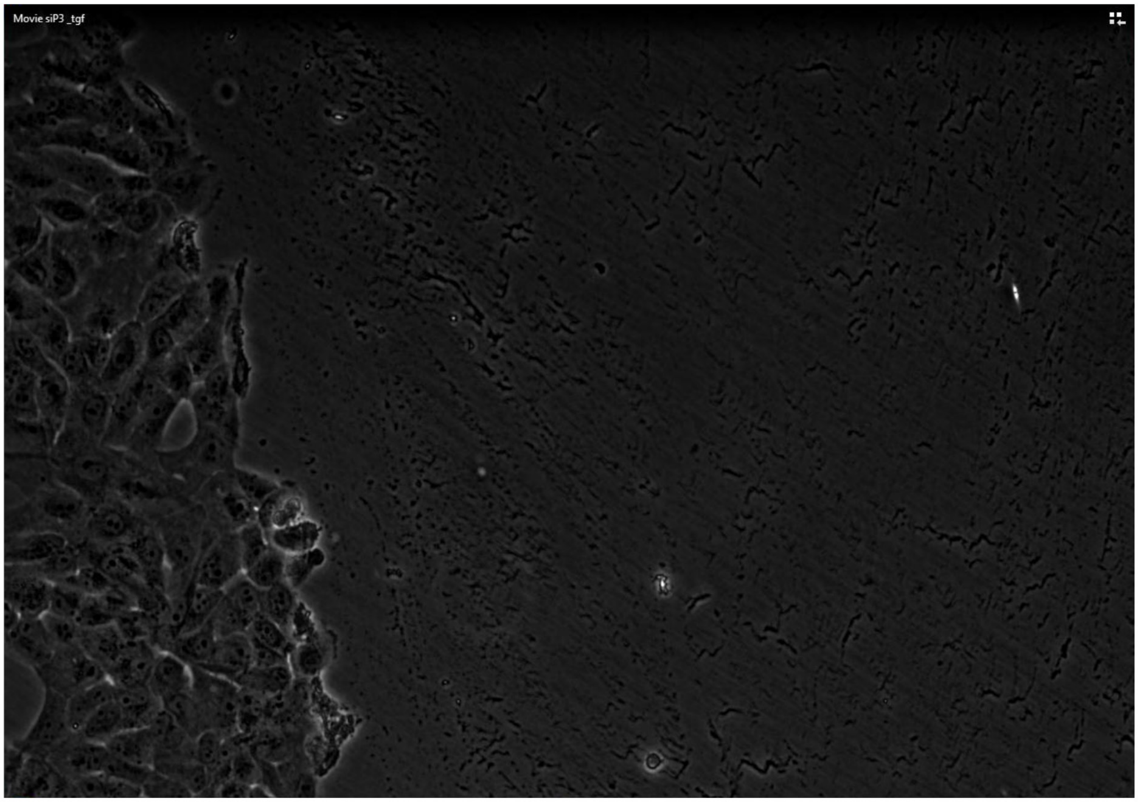

Supplementary Movie S1: PARP3 depletion slows down the migration of TGFβ-treated MCF10A cells in scratch wound closure assays *in vitro*. Confluent TGFβ-treated control (scr) and PARP3-depleted (siPARP3) MCF10A cells were artificially wounded and pictures of the wound closure were taken every 20 minutes for 48 hours. Images were compiled, and movies were created using the software Image J.

REFERENCES

1. Boehler C, Gauthier LR, Mortusewicz O, Biard DS, Saliou JM, Bresson A *et al*. Poly(ADP-ribose) polymerase 3 (PARP3), a newcomer in cellular response to DNA damage and mitotic progression. Proc Natl Acad Sci USA 2011; 108: 2783-2788.
2. Barretina J, Caponigro G, Stransky N, Venkatesan K, Margolin AA, Kim S *et al*. The Cancer Cell Line Encyclopedia enables predictive modelling of anticancer drug sensitivity. Nature 2012; 483: 603-607.
3. Tan TZ, Miow QH, Miki Y, Noda T, Mori S, Huang RY *et al*. Epithelial-mesenchymal transition spectrum quantification and its efficacy in deciphering survival and drug responses of cancer patients. EMBO Mol Med 2014; 6: 1279-1293.
